# Biodegradable Magnesium Alloys Promote Angio‐Osteogenesis to Enhance Bone Repair

**DOI:** 10.1002/advs.202000800

**Published:** 2020-06-23

**Authors:** Hyung‐Seop Han, Indong Jun, Hyun‐Kwang Seok, Kang‐Sik Lee, Kyungwoo Lee, Frank Witte, Diego Mantovani, Yu‐Chan Kim, Sion Glyn‐Jones, James R. Edwards

**Affiliations:** ^1^ Center for Biomaterials, Biomedical Research Institute Korea Institute of Science and Technology Seoul 02792 Republic of Korea; ^2^ Botnar Research Centre, Nuffield Department of Orthopaedics, Rheumatology and Musculoskeletal Sciences (NDORMS) University of Oxford Oxford OX3 7LD UK; ^3^ Environmental Safety Group Korea Institute of Science & Technology Europe Saarbrücken 66123 Germany; ^4^ Biomedical Engineering Research Center, Asan Institute for Life Sciences Asan Medical Center, College of Medicine, University of Ulsan Seoul 05505 Republic of Korea; ^5^ Department of Prostodontics, Geriatric Dentistry and Craniomandibular Disorders Charité‐Universitätsmedizin Berlin Berlin 14197 Germany; ^6^ Laboratory for Biomaterials and Bioengineering, CRC‐I, Dept. Min‐Met‐Materials Engineering & CHU de Québec Research Center Laval University Quebec G1V 0A6 Canada

**Keywords:** angiogenesis, biodegradable metals, osteogenesis

## Abstract

Biodegradable metallic materials represent a potential step‐change technology that may revolutionize the treatment of broken bones. Implants made with biodegradable metals are significantly stronger than their polymer counterparts and fully biodegradable in vivo, removing the need for secondary surgery or long‐term complications. Here, it is shown how clinically approved Mg alloy promotes improved bone repair using an integrated state of the art fetal mouse metatarsal assay coupled with in vivo preclinical studies, second harmonic generation, secretome array analysis, perfusion bioreactor, and high‐resolution 3D confocal imaging of vasculature within skeletal tissue, to reveal a vascular‐mediated pro‐osteogenic mechanism controlling enhanced tissue regeneration. The optimized mechanical properties and corrosion rate of the Mg alloy lead to a controlled release of metallic Mg, Ca, and Zn ions at a rate that facilitates both angiogenesis and coupled osteogenesis for better bone healing, without causing adverse effects at the implantation site. The findings from this study support ongoing development and refinement of biodegradable metal systems to act as crucial portal technologies with significant potential to improve many clinical applications.

## Graphical abstract

An integrated state‐of‐the‐art in vivo and in vitro approach reveals clinically approved Mg5Ca1Zn samples stimulate accelerated bone healing by releasing anabolic metallic ions into the surrounding tissues to enhance the growth of type H blood vessels, which localize at active sites of bone remodeling and actively recruit Osterix‐positive osteoprogenitors to the degrading implant site.

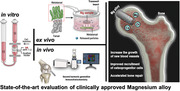

## 1 Introduction

The skeletal system consists of nonmineralized and mineralized connective tissues which enable dynamic movement and provides structural support for our body. This complex heterogeneous environment is dependent upon the strict coordination of multiple cell types (e.g., osteoblasts, osteoclasts, endothelial cells) to sustain the constant remodeling process of bone formation and resorption over a lifetime, but also to redirect cell populations during periods of bone repair and regeneration.^[^ ^1^ ^]^ Bone healing following trauma reinitiates many cellular processes of embryonic skeletal development such as the stimulation of vasculogenesis. This allows for the accumulation of mesenchymal stem cells (MSCs) which differentiate either directly into bone‐forming osteoblasts or instead to chondrocytes which first develop a cartilagenous scaffold around the healing bone and upon which new organized bone might be deposited.^[^ ^2^ ^]^

Recent studies show that both of these essential bone formation processes are coupled with the formation of blood vessels^[^ ^3^ ^]^ where proangiogenic factors like vascular endothelial growth factor (VEGF) secreted from bone cells, activates VEGF receptors on endothelial cells and precursors, but also upon chondrocytes, osteoblasts, and osteoclasts.^[^ ^4^ ^]^ Complementarily to these events, bone endothelial cells promote the formation of chondrocytes and osteoblastic cells, and control the differentiation and maintenance of hematopoietic stem cells in the bone marrow.^[^ ^5^ ^]^ Collectively, this osteo‐angiogenic relationship plays a significant role in the bone fracture healing process.^[^ ^6^ ^]^ However, the impact of skeletal implants upon this system is largely understudied.

Maintaining optimal stability during bone healing is necessary for efficient bone repair and the implantation of metal and polymer structures within and around injured skeletal elements is a common surgical approach worldwide. Successful implant materials such as titanium offer strong structural support, but the inert nature offers poor osteointegration and biocompatibility. Conversely, the variety of polymer materials available offer improved biological properties and biodegradation at the expense of reduced structural support. Recent advances in alloy development have produced metallic implants that biodegrade in situ.^[^ ^7^ ^]^ These include novel clinically approved magnesium alloys such as extruded Mg5Ca1Zn (Mg5Ca1Zn), which offer strength during the bone healing period after which they fully degrade within the skeletal microenvironment.^[^ ^8^, ^9^ ^]^ Importantly, here we show the potential for accelerated bone healing stimulated by the release of constituent ions and a direct cellular mechanism through which improved bone healing occurs, using a combined in vitro, ex vivo, and in vivo approach employing advanced microscopy techniques and 3D modeling of high‐resolution CT scans.

## 2 Results and Discussion

**Figure 1:**
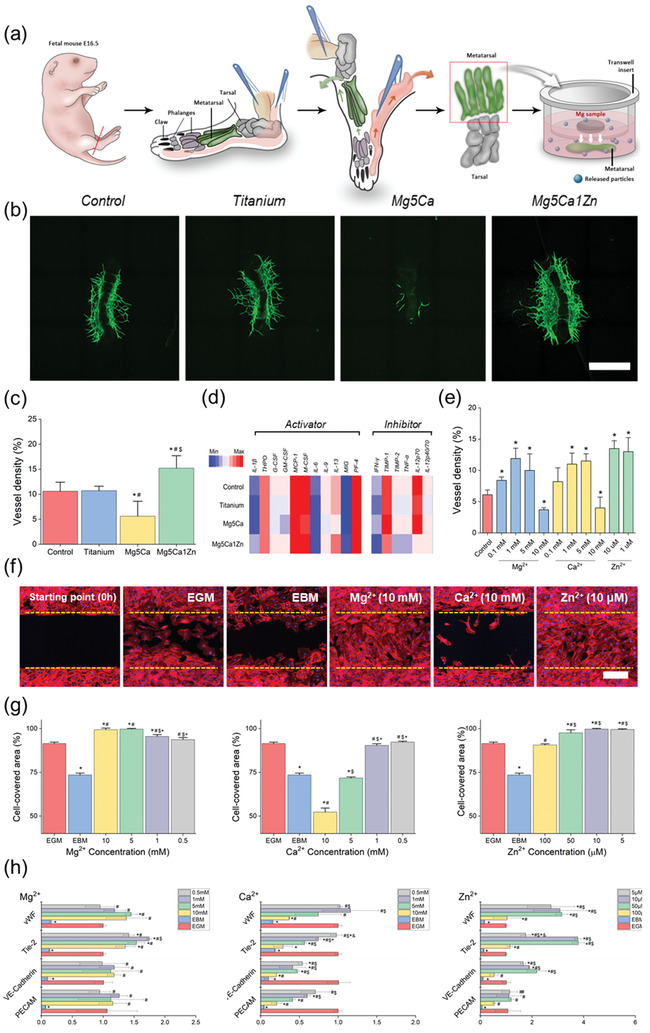
a) Schematic illustration of fetal mouse metatarsal assay culture method. b) Representative fluorescent images of CD31 positive vessel outgrowth from metatarsal growth under different types of metal alloys. c) Quantified vessel density after 5 days of culture in alpha‐MEM treated with different types of biodegradable metals. “*,” “#,” and “$” indicate significant differences compared to Control, Titanium, and Mg5Ca, respectively (*p* < 0.05). Scale bar, 500 µm. d) Heat map of angiogenic cytokine expression. e) Quantified vessel density after 5 days of culture in alpha‐MEM treated with different concentration of Mg, Ca, and Zn ions. f) Scratch assay test on HUVEC treated with Mg, Ca, and Zn ions at 16 h postinjury. Scale bar, 200 µm g) Cell migration evaluated by repopulation of the cleared area with cells; the cell covered area (%) was measured by WimScatch (a web‐based software from Wimasis). h) Quantification analysis of CD31, VE‐Cadherin, Tie‐2, and vWF in Mg‐, Ca‐, and Zn‐ ion treated HUVEC using real‐time RT PCR. “*,” “#,” “$,” and “+” indicate significant differences compared to EGM, EBM, 10, 5, 1 × 10^−3^ m, respectively for MgCl_2_ and CaCl_2_ treatment group. “*,” “#,” “$,” and “+” indicate significant differences compared to EGM, EBM, 10 × 10^−6^, 5 × 10^−6^, 1 × 10^−6^ m, respectively for ZnCl_2_ treatment group.

Angiogenesis, which involves new blood vessels sprouting from existing capillaries, plays a crucial role in bone development and in the healing process. The fetal mouse metatarsal assay allows the observation and quantitation of sprouting blood vessels in an ex vivo microenvironment and more closely mimics the in vivo formation of natural blood vessels than any other assay available^[^ ^10^ ^]^ (**Figure** **1**a). The growth of vessels from isolated embryonic metatarsals captures the key aspects of natural blood vessel formation such as endothelial cell lumen formation, tip cell development, and recruitment of adjacent support cells.^[^ ^11^ ^]^ Since the stimulation of vessel growth from the metatarsal is guided primarily by endogenous factors, the accurate evaluation of the angiogenic effect of various components upon this process can be assessed. Furthermore, the development of a complex network of blood vessels in a 2D plane allows quantitative analysis of growth.^[^ ^12^ ^]^

Biodegradable metal alloy and inert titanium samples were immersed in alpha‐MEM using transwell inserts to study in co‐culture, the direct effect of biodegradation materials on angiogenesis through the fetal mouse metatarsal assay. As shown in Figure 1b,c, released metallic ions from biodegradation of Mg5Ca1Zn samples^[^ ^9^ ^]^ significantly induced angiogenesis, demonstrating a web‐like complex of blood vessel structures in the cultured metatarsal. This is due to the slow corrosion rate of Mg5Ca1Zn samples (Figure S1, Supporting Information), which leads to release of Mg, Ca, and Zn ions at sustained concentrations that facilitate more rapid growth of new blood vessels. Media cultured with titanium samples showed a similar response to the control sample cultured without any metal samples, due to the corrosion resistant nature of titanium. Titanium remains intact during the entire period of metatarsal culture and does not appear to release any metallic particles into the environment, which might improve angiogenesis. In contrast, angiogenesis was significantly inhibited from metatarsals cultured in media containing the Mg5Ca alloy samples. Rapid corrosion of the Mg5Ca (Figure S1, Supporting Information) in alpha‐MEM results in an increase in pH level and release of higher concentrations of metallic ions into the culture system. These data indicate that the moderate degradation profile of the Mg5Ca1Zn alloy is not toxic to the growth of blood vessels in the ex vivo environment and in fact, stimulates enhanced vasculogenesis through the optimal release of metallic ions in quantities which appear proangiogenic.

In addition to visualizing vasculogenesis, the metatarsal assay allows for extraction of RNA and proteins from the primary vessel outgrowth. The influence of degrading biodegradable Mg alloys on the proangiogenic secretory profile was investigated in protein lysates using a cytokine array consisting of angiogenic activators and inhibitors. A normalized heat map of protein expression to cell number (derived from blood vessel density analysis) showed an increase in expression of proangiogenic proteins from metatarsals cultured with Mg5Ca1Zn samples, such as IL‐1*β*, GM‐CSF(as shown in Figure 1d), along with decreased expression of angiogenic inhibitors (e.g., IFN*γ*, TNF*α*) when compared to control and titanium groups. However, metatarsals cultured with Mg5Ca samples showed decreased expression of angiogenic activator levels and a higher secretion of angiogenic inhibitors. No significant difference was observed between Control and titanium groups. Overall, protein expression from primary vessels following culture with implant materials correlated closely with that of blood vessel density analysis and was in agreement with in vitro assays (summarized in Figure 1f,h).

The scratch assay is a well‐established method to measure cell motility over time. Cell migration is observed as the wound from the scratch is closed by the proliferation and active movement of Human Umbilical Vein Endothelial Cells (HUVEC) (as shown in Figure 1f and Figure S2 (Supporting Information)). As expected, enhanced migration and wound closure were observed using untreated endothelial growth medium (EGM) as positive control. Endothelial basal medium (EBM) was used as a negative control environment to illustrate the slower rate of cell migration and cell proliferation. Faster cell migration and wound closure rates from Mg treated cells were observed for concentrations higher than 1 × 10^−3^ m when compared to the positive control group. Similar to the results from proliferation assays, HUVEC migration was decreased or inhibited in the presence of Ca ions when compared to the positive control after 16 h. The results from scratch assays of HUVECs treated with 10 × 10^−3^ m Ca were consistent with that of negative control cultures. HUVECs exposed to Zn at a concentration of 50 × 10^−6^ m showed a drastic increase in cell migration on the scratch‐wound healing assay after just 8 h. Consequently, wound closure was almost completed at 16 h in the presence of 50 × 10^−6^ m Zn. Interestingly, the higher concentration of 100 × 10^−6^ m Zn treatment did not further accelerate the migration rate of the HUVEC.

HUVEC cultures treated with Mg‐ion exhibited similar or higher expression of proangiogenic genes (VE‐cadherin, vWF, and Tie‐2) compared to untreated (EGM only) or negative control (EBM only) at 72 h, as shown in Figure 1h. For example, HUVECs treated with 10 × 10^−3^ m Mg ion had a higher expression of endothelial marker genes compared with control groups (3.90 ± 0.47‐fold for CD31, 1.82 ± 0.03‐fold for VE‐Cadherin, 1.37 ± 0.22‐fold for Tie‐2 and 2.10 ± 0.01‐fold for vWF compared to EGM). HUVECs treated with Ca‐ion exhibited decreased proangiogenic gene expression (Figure 1h). When compared to the expression of each gene in the control groups (EGM only), a significantly reduced level of expression was observed from HUVECs cultured in the presence of Ca ion. For example, 10 × 10^−3^ m Ca ion had a lower expression of endothelial marker genes compared with control groups (0.21 ± 0.07‐fold for CD31, 0.20 ± 0.01‐fold for VE‐Cadherin, 0.29 ± 0.12‐fold for Tie‐2 and 0.37 ± 0.01‐fold for vWF compared to EGM). The HUVECs, treated with Zn‐ion exhibited similar or higher expression of proangiogenic genes compared to untreated (EGM only) or negative control (EBM only) at 72 h, as shown in Figure 1h. For example, 50 × 10^−6^ m Zn ion treatment increased endothelial gene expression 1.19 ± 0.11‐fold for CD31, 4.18 ± 0.80‐fold for VE‐Cadherin, 7.57 ± 0.46‐fold for Tie‐2 and 4.46 ± 0.40‐fold for vWF, compared to EGM.

To more accurately control for and assess the effect of individual alloy ion concentrations upon primary vessel outgrowth, known concentrations of Mg, Ca and Zn ions were added to metatarsal cultures for 5 days. As shown in Figure 1e, Mg ions facilitated the growth of new blood vessels when treated with 0.1 × 10^−3^, 1 × 10^−3^, and 5 × 10^−3^ m of Mg ions (versus no ion control). Interestingly, 10 × 10^−3^ m of Mg ions reduced the ex vivo formation of new primary blood vessels, a feature which was not observed in in vitro assays of HUVEC cells. The addition of Ca ions to primary cultures increased vessel density after 5 days of culture up to 5 × 10^−3^ m, which was in close agreement with HUVEC proliferation results (Figure S3, Supporting Information), whilst 10 × 10^−3^ m Ca ions greatly reduced the density of blood vessels in both assays. The highest vessel density was achieved using 1 × 10^−6^ and 10 × 10^−6^ m Zn ions in both assays with 10 × 10^−6^ m Zn ions increasing vessel density by two‐fold when compared to control. However, the addition of higher concentrations of Zn ions did not further improve this response.

**Figure 2:**
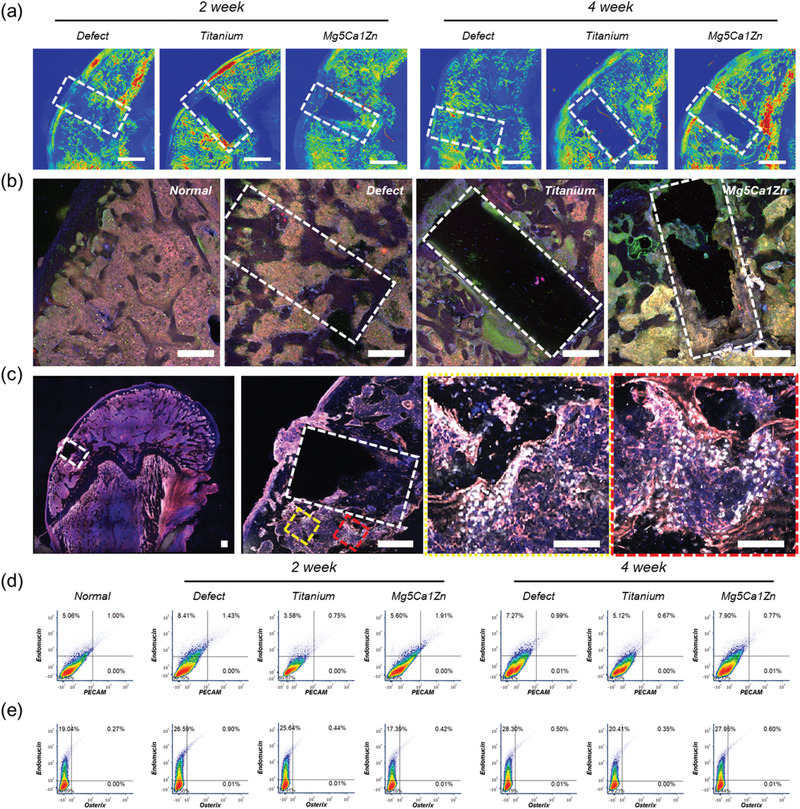
a) SHG images of normal SD rat femur, empty defect site, titanium, Mg5Ca1Zn samples after 2 and 4 weeks postoperation. Scale bar represents 1 mm in length and white dash lines represent the original defect created during surgery. Green and Red colors represent the increasing concentration of collagens detected by the SHG. b) Confocal microscopy images of immunostained sections from normal bone, empty defect site, titanium, Mg5Ca1Zn samples after 4 weeks postoperation (Endomucin—Red, Osterix—Green, DAPI—Blue). Scale bars, 500 µm. c) High resolution confocal microscopy images of immunostained sections Mg5Ca1Zn samples after 4 weeks postoperation (Endomucin—Red, Osterix—White, DAPI—Blue). Scale bar, 500 µm. Yellow and red dotted square areas indicate magnified regions with scale bars 200 µm. d) Flow cytometry dot plots showing expression of PECAM and Endomucin from the cells collected from femoral head of SD rats with empty defect site, Titanium or Mg5Ca1Zn after 2 and 4 weeks postoperation. e) Flow cytometry dot plots showing expression of Osterix and Endomucin from the cells collected from femoral head of SD rats with empty defect site, Titanium or Mg5Ca1Zn after 2 and 4 weeks postoperation.

It is a necessary feature of implanted bone fixation devices that normal, routine mechanical loading must be sustained without causing a severe immune response, to permit new bone growth at the fracture site to proceed adequately. For biodegradable materials, a slow degradation rate is required to allow such integration of the implant to the surrounding tissue. Rapidly degrading magnesium alloys such as Mg5Ca, lead to release of hydrogen gas and metallic ions into the local physiological environment at a rate that is not beneficial for normal fracture healing. As shown in Figure S4 in the Supporting Information implantation of a fast degrading Mg alloy in the femoral condyle of SD rats resulted in insufficient healing of the bone defect site. It also revealed the formation of low‐density tissue next to the implant clearly identifiable as a different color when compared to the confirmed empty spaces, suggesting the possibility of diseased fibrotic tissue formation. Fibrotic disease at the implantation site normally occurs when the wound healing process fails to complete adequately and where a typical immune response targeting the foreign materials is initiated. Such a response hinders the early stages of bone regeneration, including collagen production and osteoid formation. Identifying collagen deposition in tissue sections containing biodegradable metal by commonly used immunohistochemical staining, results in active corrosion of the implanted metal by the staining solution and leads to the generation of hydrogen gas bubbles which hinders the accurate analysis of the collagen structure. Recently, a technique to obtain thick cryo‐sections for high resolution 3D imaging of bone was introduced by Kusumbe et al.^[^ ^13^ ^]^ This method was adapted to obtain thick tissue sections of femoral condyle implanted with Mg samples for second harmonic generation (SHG) imaging.^[^ ^14^ ^]^ At 4‐week postoperation, SHG imaging of Mg5Ca1Zn alloy implanted into femoral condyles of SD rats, showed abundant collagen deposition on top of the implant sample (**Figure** **2**a). The even distribution and high intensity of the SHG signals confirm the abundance of collagen adjacent to Mg5Ca1Zn alloy samples. During bone formation and healing, osteoblasts synthesize and initially deposit type I collagen in random directions. Such randomly deposited and mineralized collagen fibers form the basis of temporary, woven bone seen in the initial fracture callus which is significantly lower in strength than the final lamellar bone structure which has aligned, organized collagen fibrils and mineralized osteoid. In order to study the efficacy of Mg5Ca1Zn samples further, implanted bone images were compared to the SHG image of titanium samples and empty defect sites created in the femoral condyle of SD rats after 2 and 4 weeks. After 2 weeks of implantation, SHG images of biodegradable Mg5Ca1Zn samples showed good connection with the surrounding tissues without empty void spaces and abundance of collagen at the implantation site. Titanium implanted into bone is known to result in osteointegration which allows direct apposition of new bone on the surface of the titanium implant. The cortical bone was not completely closed for all of the tested group after 2 weeks. The empty defect site was not yet replaced with new bone completely and structure of the collagen near Mg5Ca1Zn resembled that of the empty defect site more closely than that of titanium samples. This could be related to the active biodegradation of Mg5Ca1Zn which is in good agreement with the our previous clinical trial study^[^ ^9^ ^]^ showing a possible absence of cancellous bone and atypical shade of surrounding bone at the implant interface at the early stage of implantation and healing (2‐4 weeks after surgery). All 53 cases reported in clinical trial study^[^ ^9^ ^]^ showed fracture reunion within ≈4–6 weeks and this again is in good agreement with the results after 4 weeks. After 4 weeks of implantation, the cortical bone was completely closed for all the groups tested and where Mg5Ca1Zn alloy showed similar SHG signals to those from bioinert titanium implants. Thick cortical bone was formed on top of the Mg5Ca1Zn alloy without the voids observed in fast corroding Mg samples. This result clearly shows the direct deposition of collagen matrix on the surface of degrading Mg5Ca1Zn and growth of new bone tissue around the degrading Mg5Ca1Zn samples which is equivalent with the clinically proven titanium samples. Utilization of SHG allowed observation of collagen structures adjacent to the biodegradable alloy without the creation of new hydrogen gas bubbles from histological staining procedures and showed that the newly developed Mg5Ca1Zn alloy facilitates high levels of direct collagen deposition, a prerequisite for new bone formation.

The adjacent cryo‐sectioned slide obtained after sectioning of the thick metal containing slide used for SHG was prepared for immunohistological evaluation. During bone formation and repair, mesenchymal stem cells (MSCs) differentiate into osteoblast precursors and mature bone‐forming cells required for fracture healing. Importantly, the growth of new blood vessels is coupled with osteoprogenitor invasion during bone deposition^[^ ^3^, ^15^ ^]^ and where the recently described H‐ and L‐type vasculature offer distinct functional roles. Type H blood vessels are relatively small in quantity, show high expression of CD31 and Endomucin and have been deemed the building block for new bone, found mostly in actively remodeling sites. Interestingly, Osterix‐positive osteoprogenitors are found to be positioned selectively around the type H blood vessels and not L blood vessels.

As shown in Figure 2b,c, immunofluorescent staining of normal bone sections shows blood vessel structures positive for Endomucin and scattered Osterix‐positive cells. A greater number of Osterix‐positive osteoblasts are observed at the empty defect site and are more densely located where active bone regeneration of the bone defect is occurring. Interestingly, femurs implanted with titanium showed almost cortical‐like dense bone layers formed on the surface. Significantly increased numbers of Osterix‐positive cells were found in these dense new bone layers when compared to the regions distant from the implant site. Implanted Mg5Ca1Zn samples showed a similar pattern of cell distribution to titanium samples where significantly increased numbers of Osterix‐positive cells were located near the implantation site. The creation of densely packed bone near the implant was not observed for Mg5Ca1Zn samples indicative of the actively degrading surface of the Mg5Ca1Zn alloy. Newly generated bone tissue was observed in direct contact with the degrading surface of the Mg5Ca1Zn samples, showing good biocompatibility to the material. Confocal imaging of the whole femoral head implanted with Mg5Ca1Zn alloy was performed at higher resolution to assess the overall distribution of Osterix‐positive cells and observe the active bone remodeling near the implantation site. Figure 2c shows the whole image of the immunostained femoral head section containing a Mg5Ca1Zn sample. The Mg5Ca1Zn implant was carefully removed before staining to avoid the formation of hydrogen gas bubbles. The complete image of the femoral head allows for the visualization of Osterix‐positive cell distribution around the actively remodeling sites of the bone (indicated by yellow and red dotted box). A magnified high‐resolution image of the implantation site revealed densely packed Osterix‐positive cells located around type H blood vessels which have formed near the implantation site. This result indicates that Mg5Ca1Zn samples facilitate optimal bone healing by releasing metallic ions and actively recruiting osteoprogenitors near the implant site at a degradation rate acceptable for the tissue.

The quantification of Endomucin, Osterix and CD31 (PECAM) using flow cytometry validated these outcomes (Figure 2d,e). The distribution of CD31+, Endomucin+ type H blood vessels is reported to be 1.77% in younger mice (4 weeks old) with active bone growth.^[^ ^3^ ^]^ The result from this study showed ≈1% type H blood vessels in the normal adult femoral head, which is supported by previously reported findings.^[^ ^3^ ^]^ As shown in Figure 2d, high expression of CD31 and Endomucin of 1.43%, 0.75%, and 1.91% for empty defect, titanium and Mg5Ca1Zn group respectively, was observed. The SHG imaging showed an abundance of deposited collagen matrix near the Mg5Ca1Zn implantation site after 2 weeks when compared to the titanium group. Interestingly, flow cytometry results showed a higher number of H type blood vessels within the femoral head implanted with Mg5Ca1Zn samples when compared to the titanium group. The empty defect group showed a higher number of H type blood vessels than the normal group that did not receive any surgical treatment, and titanium group showed fewer H type blood vessels. The difference relative abundance of type H blood vessels in Mg5Ca1Zn and Titanium was reduced (0.77% and 0.67% respectively). This could be related to the slower degradation rate of Mg5Ca1Zn caused by the formation of a protective magnesium hydroxide layer. At the initial stages of corrosion, there are relatively higher numbers of metallic ions released into the environment. However, as magnesium degradation progresses, a corrosion protective layer is formed to slow and stabilize the degradation rate. As demonstrated in vitro and ex vivo, released Mg, Ca, and Zn ions would increase the proliferation of endothelial cells and the growth of new blood vessels resulting in a higher number of blood vessels generated near the implantation site, leading to improved recruitment of osteoprogenitor cells and accelerated repair. This is clearly demonstrated in Figure 2e which shows higher relative abundance of Osterix‐positive cells in samples implanted with Mg5Ca1Zn after 4 weeks. The relative quantity of Osterix‐positive cells is similar for the titanium group and Mg5Ca1Zn group after 2 weeks (0.44% for titanium and 0.42% for Mg5Ca1Zn) but the difference between the two groups is nearly doubled after 4 weeks (0.35% for titanium and 0.60% for Mg5Ca1Zn). A decrease in the relative number of Osterix‐positive cells in the defect model over time is also in agreement with the findings described in this study.

Previous in vitro evaluations of our Mg alloy indicated a positive effect upon skeletal cells and bone regeneration following the release of metallic ions (Mg, Ca, and Zn). Whilst such a conventional approach allows for the observation of bone cell growth and proliferation, it does not accurately portray the 3D in vivo environment into which biodegradable metals will be applied. The corrosion profile of biodegradable metals depends heavily on the local environments and experimentation aiming to recapitulate this niche must simulate the key aspects of in vivo conditions, such as blood flow‐induced magnesium corrosion, hydrogen evolution, and local pH change, for the accurate evaluation of newly developed biodegradable materials. Since the surface of the metal is in immediate contact with blood and surrounding tissues, blood flow plays a vital role during the initial stage of implantation,^[^ ^16^ ^]^ and several studies have shown that the rate of the corrosion is increased for samples immersed in dynamic flow condition.^[^ ^17^, ^18^ ^]^ A conventional static culture system is designed for testing materials that are corrosion resistant and does not take key aspects of biodegradable materials into consideration. Furthermore, the transport of nutrients occurs only by diffusion in static culture conditions and the absence of cell culture medium circulation often results in increased nutrient and metabolite concentrations on the surface of the material which might inhibit cell migration and tissue formation.^[^ ^19^ ^]^ The lack of oxygen transport and removal of waste from a 3D scaffold in static culture also leads to a nonuniform distribution of cells and decreased proliferation and differentiation.^[^ ^20^ ^]^

Consequently, to simulate the circulation of blood to perfuse and facilitate transportation of oxygen and nutrients to the cells in 3D scaffolds, an advanced perfusion bioreactor system was developed to mimic the complex biological environment of bone. Bioreactors demonstrate several advantages over conventional 2D culture systems, mainly because they allow seeding of cells on materials under dynamic flow conditions while enhancing the transport of metabolites and catabolites throughout the 3D structure. Also, it reduces potential contamination through unnecessary handling and enables greater control of pseudo‐physiological environmental conditions like pH, oxygen, and CO_2_ concentration.^[^ ^21^ ^]^ For the accurate evaluation of orthopedic implant devices, the flow‐induced stress and resulting mechanical stimulation upon bone cells must be considered. Mechanical loading is responsible for interstitial fluid flow through canalicular and lacunar spaces in bone and such mechano‐stimulation from fluid flow influences the proliferation and differentiation of bone cells occupying these spaces.^[^ ^22^ ^]^ Furthermore, corrosion of Mg alloys in 2D culture systems leads to release of OH which in turn results in the rapid saturation of alkaline pH in the local corrosion environment, leading to excessive deposition of calcium phosphate that hinders accurate evaluation.^[^ ^7^ ^]^ Previous bioreactor studies using Mg alloys have focused on stent applications and the control of mechanical loading, fluid composition and CO_2_ concentration.^[^ ^18^ ^]^ However, studies investigating the change in corrosion within a bioreactor, effect of released metal ions from Mg alloys, change of pH level, and release of hydrogen gas upon bone cells seeded within a perfusion bioreactor have not been performed. Therefore, Mg5Ca1Zn samples were implanted on porous hydroxyapatite scaffold and press‐fitted to a perfusion chamber to investigate the direct effect of magnesium degradation on human osteoblast cells in an in vivo‐simulated environment, to provide a systematic understanding of bone cell‐magnesium alloy interaction under a flow‐induced loading condition including corrosion profile, corrosion rate, observation of bone formation markers, osteoblast proliferation and differentiation.

**Figure 3:**
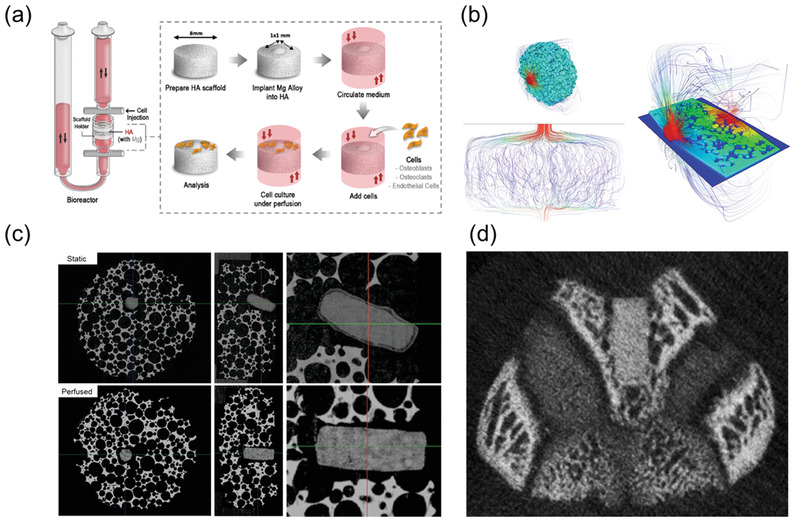
a) Schematic illustration of perfusion bioreactor and Mg sample implanted on HA scaffold b) Water molecular diffusivity simulation showing the flow of media through the open porous structure of hydroxyapatite scaffold. c) µCT comparison of Mg5Ca1Zn samples immersed in static conditions with a thick layer of corrosion by‐product on the surface of Mg and perfusion condition without accumulation of corrosion by‐product on the surface after 4 weeks. d) 4 weeks postoperation in vivo CT of femoral condyle of SD rat implanted with Mg5Ca1Zn.

The corrosion profile was compared between static and perfused culture conditions (**Figure** **3**). Typically, the corrosion of Mg alloys results in the formation and deposition of a corrosion product layer. This was observed on the surface of Mg alloy samples immersed in static conditions, while uniform corrosion with relatively flat regions were seen for the samples cultured in perfused conditions. indicating a more rapid removal of the corrosion product by the flow of medium. Unlike static culture systems, 3D cell culture techniques facilitate cell penetration into the pores of the scaffold and delivery of oxygen and nutrients throughout. Moreover, exposure to fluid shear stress enhances the viability, proliferation, differentiation, and bone forming ability of osteoblastic cells in vivo of osteoblasts when compared to conventional 2D static culture conditions.^[^ ^23^ ^]^ The simulation of media flow through the HA scaffold was modeled using Avizo software following high resolution uCT scanning using the Xradia 810 Ultra X‐ray system (Zeiss), and confirmed the pore connectivity and open porous trabecular bone‐like structure of this material (Figure 3b). The inner layer of the Mg corrosion product consists of magnesium oxide whereas the outer layer is composed of calcium phosphate.^[^ ^9^ ^]^ During the initial phase of corrosion, released Mg ions react with OH from cathode sites to form the magnesium oxide layer. An increase in local pH follows causing calcium phosphate precipitation on the surface of the corroding magnesium. The corrosion rate of Mg depends heavily on the pH level. Conventional static culture conditions without effective fluid flow leads to a higher local pH level around the alloy and favorable environment for magnesium oxide formation and calcium phosphate precipitation.^[^ ^24^ ^]^ However, this does not reflect the in vivo environment of human and animal bones where active blood flow occurs constantly at rates ranging from 5 to 20 mL min^−1^ per 100 g^[^ ^25^ ^]^ and where blood flow in the fractured tibia was shown to be 3 to 6 times higher than a normal tibia after 24 h of injury.^[^ ^26^ ^]^ In Figure 3c, a corrosion by‐product layer is absent from alloy samples cultured in perfused conditions, which can be attributed to the flow‐induced loading from the circulating media. In addition to the prevention of calcium phosphate precipitation by mechanical loading, flowing medium leads to faster ion diffusion and prevents the increase in local pH. Calcium phosphate is known to deposit faster in slowly diffusing alkaline environments and such precipitation was almost completely inhibited in perfused conditions.^[^ ^27^ ^]^ This result is consistent with findings from other studies.^[^ ^16^, ^28^ ^]^ As shown in Figure 3d, a similar corrosion profile was observed from the live µCT scan of SD rats implanted with the same Mg alloy at 4 weeks postoperation. These similarities in corrosion profile confirm the close simulation of an in vivo‐like environment and support the reliability of the perfused 3D cell culture system.

**Figure 4:**
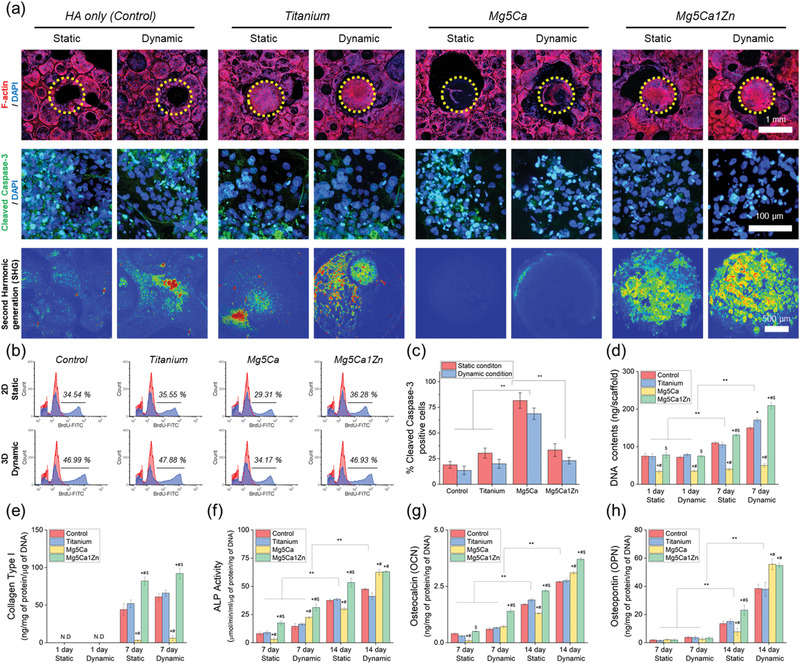
a) Cytoskeletal structures hFOBs after 24 h of culture on the HA, Mg‐based alloy implanted HA and titanium implanted HA. The images were acquired by fluorescence staining for F‐actin (red), and DAPI (nuclei). Representative images of hFOBs stained for Cleaved Caspase‐3. The images were acquired by fluorescence staining for Caspase‐3 (green), and DAPI (nuclei). Second harmonic generation (SHG) imaging of cells on metal alloy. b) FACS results of hFOB proliferation after 24 h of culture under static and dynamic conditions using control HA, HA implanted Titanium and HA implanted with two different type of Mg‐based alloys. c) Quantification of apoptosis from each sample. d) Quantification of DNA contents. e) ELISA analysis of the Collagen I content of cells on metal alloy. Quantification of production of f) ALP, g) OCN and h) OPN bone protein. “*,” “#,” and “$” indicate significant differences compared to Control, Titanium and Mg5Ca, respectively (*p* < 0.05). ** indicates significant difference (*p* < 0.05).

The use of a perfusion bioreactor significantly increased human fetal osteoblastic hFOB 1.19 cell penetration through the HA scaffold (Figure S5, Supporting Information). The hFOB 1.19 cells did not migrate down to the empty defect site created in HA after 1 day of static culture. In contrast, confocal Z stack images clearly showed migrating osteoblasts penetrating the empty defect in perfused conditions over the same time frame. To investigate the bioactivity of metal alloys, the attachment of hFOBs for both static and dynamic flow conditions were observed microscopically after 24 h of culture. As shown **Figure** **4**a, cells adhered and thrived on titanium and Mg5Ca1Zn surfaces when compared to that of Mg5Ca. As the corrosion rate of the Mg5Ca alloy is roughly 20 times faster than the Mg5Ca1Zn alloy, the rapid degradation rate might influence cell adherence on the surface of Mg5Ca. Cell attachment on Mg5Ca was slightly higher in dynamically perfused conditions but no additional benefit to cell adhesion was observed for titanium and Mg5Ca1Zn cultures.

As shown in Figure 4b, FACS analysis revealed no significant difference in hFOB proliferation between materials tested in 2D static conditions, except in the Mg5Ca group after 24 h of culture where a significant decrease was seen. This alteration in cell growth is attributed to the fast corrosion rate of the Mg5Ca alloy. There was a significant difference in osteoblast proliferation for the same materials cultured in 3D dynamic conditions compared to 2D static cultures. For example, osteoblast proliferation (BrdU intensity values) in 2D static conditions were 34.54%, 35.55%, 29.31%, and 36.28% for Control, titanium, Mg5Ca and Mg5Ca1Zn, respectively. However, a significant increase was observed in 3D dynamic conditions to 46.99%, 47.88%, 34.17% and 46.93%, respectively. To determine the level of apoptosis induced by the degradation of metal alloys, immunofluorescence staining was performed for cleaved caspase 3 (CC3), a marker of mid‐stage apoptosis. Representative images from each culture conditions are shown in Figure 4c. As expected, a significant increase in osteoblast apoptosis was observed in the Mg5Ca group. In static culture conditions, apoptotic CC3 positive cells/total number was 81.67 ± 7.58, 18.96 ± 3.28, 30.39 ± 4.91, and 33.43 ± 6.27 for Mg5Ca, Control, titanium and Mg5Ca1Zn, respectively with only a small nonsignificant decrease observed for materials in dynamic culture conditions. The proliferation of hFOBs were further analyzed by measuring DNA content after culturing for 7 days (Figure 4d). The results showed no significant difference between total DNA content for tested materials in both culture conditions at the initial 1 day time point (65.50 ± 5.35, 65.24 ± 3.02 and 69.42 ± 4.17 for Control, titanium and Mg5Ca1Zn in static culture condition; 64.30 ± 0.26, 71.99 ± 1.32, and 63.49 ± 4.04 for Control, titanium and Mg5Ca1Zn in dynamic culture). However, cells cultured in dynamic conditions showed a statistically higher quantity of DNA following 7 day culture with Control, titanium and Mg5Ca1Zn, likely due to the effects of 3D perfusion mentioned earlier. Interestingly, cells cultured on Mg5Ca1Zn in dynamic conditions for 7 days showed significantly increased proliferation when compared with static culture conditions; 212.49 ± 8.34 and 122.73 ± 1.17 in dynamic culture condition and static culture condition, respectively. It was also statistically significant when compared to control HA and HA implanted with titanium. Such results indicate that the released metal ions from the magnesium alloy have beneficial effects on proliferation of osteoblasts which is in agreement with the findings from previous studies. The cells seeded on Mg5Ca showed a dramatically decreased DNA content compared to other tested samples regardless of culture conditions (35.29 ± 3.05 and 40.09 ± 0.75 in static or dynamic culture condition, respectively).

The expression level of fibrillar collagen was higher in dynamic culture conditions for all materials tested, the highest being Mg5Ca1Zn (Figure 4e). Consistent with SHG results, the protein expression level of Collagen I by hFOBs was greater for Mg5Ca1Zn cultures compared to those on titanium and Mg5Ca alloy (88.01 ± 4.69, 54.86 ± 5.01, and 1.91 ± 1.03 for Mg5Ca1Zn, titanium and Mg5Ca, respectively). In addition, Collagen I expression was higher in dynamic culture conditions than in static culture (Figure 4e). The impact of degrading alloy material was also assessed on soluble protein markers of osteogenesis including alkaline phosphatase (ALP), osteocalcin (OCN), and osteopontin (OPN) (Figure 4f,h). A significant increase in protein expression of all markers was observed in dynamic (versus static) culture conditions on Mg5Ca1Zn following 14 days, indicative of enhanced osteogenic differentiation and osteoblast activity. Interestingly, cells on Mg5Ca showed a significantly lower expression of OPN in static culture conditions (8.23 ± 3.41 and 54.45 ± 2.77, respectively). The decreased protein expression observed on Mg5Ca alloys could be related to the higher amount of metallic ions released into the media. However, when protein expression was normalized to DNA content the ratio was significantly higher compared to control and titanium. This result suggests that the rapid corrosion of the Mg5Ca alloy reduces the rate of hFOB cell attachment and proliferation, but the increased amount of metallic ions released leads to increased production of osteogenic proteins. In addition, there was no statistical difference between control (HA only) and the bioinert titanium.

## 3 Conclusion

The use of bio‐absorbable alloys have significant potential for application in a variety of medical fields. Perhaps the most likely indications are in trauma and orthopedic surgery, where implantable plates and screws can be a source of significant cost, complication and morbidity, requiring revision or removal following implantation. In this study, several state‐of‐the‐art techniques were used to investigate osteoblast formation, activity and bone formation around the biodegradable Mg5Ca1Zn alloy, revealing a significant beneficial effect of released metal ions within the bone microenvironment, stimulating blood vessel growth which promoted improved osteogenesis. In vitro and ex vivo metatarsal assays further confirmed the positive effect of controlled Mg5Ca1Zn alloy degradation and ion release on angiogenesis, supported by SHG and immunofluorescent imaging of enhanced collagen matrix deposition at the implantation site. These findings demonstrate that Mg5Ca1Zn samples stimulate accelerated bone healing by releasing anabolic metallic ions into the surrounding tissues to enhance the growth of blood vessels and actively recruit osteoprogenitors near the implant site.

## 4 Experimental Section

##### Fetal Mouse Metatarsal Angiogenesis Assay

Fetal mouse metatarsals were dissected from E17.5 embryos of CD1 mouse. The feet were cut off just above the ankle joint of fetuses under a dissection stereomicroscope and transferred to a fresh 10‐cm culture dish containing ice‐cold dissection medium with tweezers. One pair of tweezers was inserted between the skin and the calcaneus while holding the exposed fibula with the other pair of tweezers. The first pair of tweezers was slowly advanced into the footpad while removing the skin together with phalanges to leave tarsals and metatarsals intact. Excessive connective tissues connected to the metatarsals were removed and then the metatarsals were separated. The first and fifth metatarsals were thrown out and only middle 3 metatarsals were used for culture. The isolated metatarsals were cultured in 24‐well plates with alpha‐MEM. The explants were cultured with metal alloys for 5 days using the commercially available Transwell system. After 5 days, the explants were fixed and stained with FITC‐conjugated CD31 antibody. Images were observed using confocal laser scanning microscopy (LSM 700, Carl Zeiss, Oberkochen, Germany) and quantified by the Wimasis angiogenesis analysis platform (WIMASIS, GmbH, Munich, Germany).

##### Angiogenic Cytokine Arrays (Secretome Array Analysis)

A mouse antibody angiogenesis array membrane was used following the manufacturer's instruction for the analysis of cytokine from angiogenesis of fetal mouse metatarsal bone. Briefly, the metatarsal bone was removed after 5 days of incubation and lysed using RIPA lysis buffer to obtain cell lysate proteins. 250 µg of cell lysate protein were incubated with blocked membrane overnight at 4 °C. Prepared membranes were detected after incubation with luminescence solution using Gel‐Doc image station. The band intensity was scanned and analyzed using the ImageJ plug‐in “Protein array analyzer.”

##### Migration (Scratch Assay)

An endothelial cell line, Human Umbilical Vein Endothelial Cells (HUVEC), obtained from LONZA group, was grown in Endothelial Cell Growth Medium (EGM) kit in a humidified chamber with 5% CO_2_ at 37 °C. HUVEC cells were seeded in 6‐well plates at a density of 5 × 10^4^ cells/cm^2^ and allowed to grow for 24 h at 37 °C and 5% CO_2_. A small linear scratch was created in the confluent monolayer by gently scraping with sterile p200 pipette tips (care was taken during scratching process to ensure universal size and distant was made for all samples). Cells were extensively rinsed with PBS to remove cellular debris before adding the media with different treatment solution (methanolic, ethanolic and aqueous extracts) at a concentration of 50 µg mL^−1^. EBM (basal media without endothelial growth factor) was used as a negative control group. After 8 and 16 h, images of migrated cells were taken using time‐lapse microscopy to observe the closure of wound area.

##### Gene Expression

The total RNA of each cell was extracted, concentrated, purified and used to synthesize cDNA using a Maxime RT premix kit (Intron Biotechnology, Korea) according to the manufacturer's instructions. The real‐time RT‐PCR data were analyzed using the comparative threshold cycle (Ct) method. The relative expression of each gene was calculated by comparison with the expression level of GAPDH and subsequently normalized by the value for cells cultured on the control surfaces. The following oligonucleotide primer sequences were used to amplify each target sequence: GAPDH (forward, 50‐AGGGGGCAGAGATGATGACC‐30; reverse, 50‐ CAAAGGCTGAGAACGGGAAGC‐30), PECAM (CD‐31, forward, 50‐ATTGCAGTGGTTATCATCGGAGTG‐30; reverse, 50‐ CTCGTTGTTGGAGTTCAGAAGTGG‐30), VE‐Cadherin (forward, 50‐ CCTTCCCTTCCCTTCCTCTT‐30; reverse, 50‐TGGGAGCATACAGACTGGGA‐30), vWF (forward, 50‐AGCCGCATGACTGTTCTTTG‐30; reverse, 50‐ AGTTTGCAGGAAAAGGGGAA‐30). and Tie‐2 (forward, 50‐ AGGATGGAGTGAGGAATGGC‐30; reverse, 50‐ AGAGGGCATCAAAAGGCTGT‐30). Real‐time RT‐PCR using SYBR green master mix was performed with an AB 7500 sequence detection system (Applied Biosystems, Foster City, CA, USA) (40 cycles, melting at 95 °C for 15 s, annealing and extension at 60 °C for 60 s).

##### Surgical Preparation of In Vivo Model

All in vivo studies performed for this study have been reviewed and approved by the institutional animal care and use committee of Asan Medical Center. The cylinder‐shaped implant samples made out of Titanium, Mg5Ca and Mg5Ca1Zn were prepared for the in vivo experiment. The size of the samples was kept same as the in vitro experiments (1 mm in diameter and 2 mm in length). Following the approval from the Animal Care and Use Committee of Asan Medical Center in Seoul, samples were implanted into femoral condyle of Sprague Dawley (SD) rats for 2 and 4 weeks (*n* = 4 per implant sample). 6 weeks old SD rats were purchased (OrientBio) and given a week for habituation before the surgical procedure. For anaesthesia, zoletil (10 mg kg^−1^; Virbac SA) and xylazine (5 mg kg^−1^; Bayer Korea) were mixed at 2:1 ratio and injected. Minimally invasive incisions were made on the femoral condyle of SD rats, followed by defect creation of 1 mm in diameter and 2 mm in depth with an electric drill. 0.9% sterile saline was used to clean the defect and appropriate samples were implanted. Incision was sutured with 5‐0 black silk and sterilized daily with antiseptic. For a week following the surgery, cefazolin (20 mg kg^−1^) and ketorolac (1 mg kg^−1^) were injected daily as antibiotic and anti‐inflammatory therapy.

##### Sample Preparation for Second Harmonic Generation and Immunohistochemistry

The preparation of samples for immunohistochemistry was performed following the protocols published by Kusumbe et al (2014). Briefly, freshly dissected femur of SD rats were fixed immediately in ice‐cold 4% paraformaldehyde solution for 4 h. Bones were then washed three times with PBS and decalcified in 0.5 M EDTA at 4 °C with constant shaking. For cryoprotection, fully decalcified bones were immersed into cryoprotectant solution made with 20% sucrose and 2% polyvinylpyrrolidone (PVP) solution for 24 h before embedding. Embedding of tissues were done with 8% gelatin (porcine) solution mixed with 20% sucrose and 2% PVP. Cryo‐section of embedded tissue was performed for both second harmonic generation and immunohistolgy using low profile diamond coated blades on a Leica CM1850 cryostat.

##### Second Harmonic Generation

SHG imaging was performed using a dedicated two‐photon confocal microscope. Cryo‐sectioned samples were covered with 1.5 coverslip and imaged using a Zeiss Examiner Z1 two‐photon excitation laser scanning confocal microscope (Carl Zeiss, Jena, Germany) coupled to a Coherent Chameleon titanium:sapphire laser (Coherent, Glasgow, UK). The excitation wavelength was 840 nm.

##### Immunohistochemistry

Cryo‐sectioned tissues were air‐dried for 20 min and permeabilized for 10 min in 0.3% Triton X‐100. Sections were blocked for 30 min at room temperature in 5% donkey serum. The primary antibodies were mixed with 5% donkey serum in PBS for overnight at 4 °C. The following antibodies were used for this study: Endomucin (sc‐65495, Santa Cruz, diluted 1:100), PECAM‐1 conjugated to Alexa Fluor 488 (FAB3628G, R&D Systems, 1:100), PECAM‐1 (553 370, BDPharmingen, 1:100), Osterix (sc‐22536‐R, Santa Cruz, 1:200). After incubation in primary antibody solution, sections were washed three times with PBS and stained with secondary antibodies coupled with Alexa Fluor (1:400, Molecular Probes) for 1 h at room temperature. Sections were thoroughly washed with PBS before mounting with DAPI containing mounting medium and the coverslips were sealed with nail polish. The observation of immunofluorescent staining were made at high resolution with a Zeiss laser scanning confocal microscope, LSM‐710.

##### Cell Sorting by Flow Cytometry

Collected femurs were thoroughly cleaned and only the femoral head regions were used. Femoral heads were crushed with mortar and pestle in PBS at 4 °C. Whole bone marrow from the crushed bone was processed with collagenase incubation for 20 min at 37 °C. Same number of cells were stained with appropriate antibody for 45 min and cells were acquired and analysed on a BD Fortessa after washing (BD Bioscience).

##### Cell Culture in Bioreactor

Human fetal osteoblast (hFOB 1.19) was purchased from American Type Culture Collection. The growth medium used for osteoblasts was a 1:1 mixture of Ham's F12 Medium and Dulbecco's Modified Eagle's Medium, supplemented with 10% fetal bovine serum and 1% penicillin streptomycin. Cells were cultured in incubator at temperature of 34 °C, 95% humidity and 5% CO_2_. For osteogenic differentiation, osteoblasts were maintained at 37 °C in the osteogenic growth medium with 50 µg mL^−1^ Ascorbic acid, 10 × 10^−3^ m *β*‐glycerophosphate and 100 × 10^−9^ m Dexamethasone. Cells (2 × 10^6^) suspended in 1 mL of media were seeded on hydroxyapatite in the perfusion chamber and the final volume of media in reactor was 9 mL. Cells were cultured in the chamber at perfusion rate of 0.3 mL min^−1^ and the media was changed every 3 days.

##### Adhesion of Osteoblasts on Hydroxyapatite (HA)

Cells (1 × 10 ^6^ cells/HA) were seeded and cultured throughout the HA at perfusion‐based bioreactor system at a speed of 100 µm s^−1^. Cells on HA were fixed with 4% paraformaldehyde and permeabilized in cytoskeleton buffer (0.5% Triton X‐100). Following permeabilization, the HAs were blocked by incubating with 2% fetal bovine serum and 0.1% Tween‐20 in PBS and then sequentially incubated with rhodamine‐phalloidin and 4′,6‐diamidino‐2‐phenylindole (DAPI), respectively.

##### Immunofluorescence Staining for Cleaved Caspase‐3 as an Apoptosis Marker

To obtain quantitative level of apoptosis of cells on HA, cells were seeded with densities of 1 × 10^6^ cells on HA and cultured in growth medium for 24 h. Cells on the HAs were stained for Cleaved Caspase‐3, which was visualized using confocal Microscopy (LSM700). Cells on HA were fixed with 4% paraformaldehyde blocked by incubating with 2% fetal bovine serum and 0.1% Tween‐20 in PBS and then sequentially incubated with Anti‐Cleaved Caspase‐3 (1:100).

##### Fluorescence‐Activated Cell Sorting for Cell Proliferation

To evaluate proliferation at designated time point (24 h), osteoblasts were incubated with 10 × 10^−6^ m 5‐ethynyl‐2‐deoxyuridine (EdU) in growth medium for 2 h, then sequentially trypsinized and washed. Fixation (4% paraformaldehyde for 15 min), saponin‐based permeabilization and the Click‐It reaction for AlexaFluor488 labelling were performed using the Click‐iT EdU Alexa Fluor 488 Flow Cytometry Assay Kit (Life Technologies). Cells were resuspended with saponin‐based permeabilization solution and analysed on a BD Fortessa (BD Bioscience). To measure the DNA content at designated time points (1, 3, and 5 days under growth medium), osteoblasts cultured HA (*n* = 3) were lysed in RIPA lysis buffer (Santa Cruz Biotechnology). The DNA contents were measured using Quant‐iT PicoGreen dsDNA Assay Kit (Thermo Fisher Scientific) and a spectrofluorometer (FLUOstar Omega, BMG Labtech) at excitation and emission wavelengths of 480 nm and 520 nm, respectively.

##### Assaying the Levels of Differentiated Osteoblasts

Osteogenic differentiation of cells on HA was determined by Collagen type I (Col I), Alkaline phosphate (ALP), osteocalcin (OCN), and osteopontin (OPN) production by ELISA kits. Briefly, cells were seeded with densities of 1 × 10^6^ cells on HA and cultured in growth medium for 24 h. After 24 h, medium was replaced by osteogenic differentiation medium. At each time point, cells were lysed using RIPA lysis buffer with protease inhibitor added. The buffer was then collected from the samples and centrifuged at 10 000 g for 10 min. The supernatant was collected and used for the ELISA tests. The level of collagen synthesis, ALP production, secretion of OCN and OPN were analyzed using a Collagen Type I (NOVOS), ALP (Abcam), OCN (Invitrogen) and OPN (eBioscience) enzyme‐linked immunosorbent assay (ELISA) kit, respectively, according to the manufacture's instruction. The protein concentration was measured using a microBCA assay. All osteogenic differentiation activities were normalized to protein concentration of the cell lysate.

## Conflict of Interest

The authors declare no conflict of interest.

## Supporting information

Suppoorting Information
