# Supplementary material for: Biodegradable Magnesium Alloys Promote Angio‐Osteogenesis to Enhance Bone Repair
Source: Adv Sci (Weinh). 2020 Jun 23;7(15):2000800. doi: 10.1002/advs.202000800 (PMC7404158; doi:10.1002/advs.202000800)
Supplement: Supplementary file 1 — Suppoorting Information [file ADVS-7-2000800-s001.pdf]

© 2020 Wiley-VCH GmbH

ADVANCED SCIENCE
Open Access

Supporting Information

for *Adv. Sci.*, DOI: 10.1002/advs.202000800

Biodegradable Magnesium Alloys Promote Angio-Osteogenesis to Enhance Bone Repair

Hyung-Seop Han, Indong Jun, Hyun-Kwang Seok, Kang-Sik Lee, Kyungwoo Lee, Frank Witte, Diego Mantovani, Yu-Chan Kim, Sion Glyn-Jones*, James R. Edwards**

Copyright WILEY-VCH Verlag GmbH & Co. KGaA, 69469 Weinheim, Germany, 2018.

Supporting Information

Biodegradable Magnesium Alloys Promote Angio-Osteogenesis to Enhance Bone Repair

Hyung-Seop Han, Indong Jun, Hyun-Kwang Seok, Kang-Sik Lee, Kyungwoo Lee, Frank Witte, Diego Mantovani, Yu-Chan Kim, Sion Glyn-Jones*, James R. Edwards**

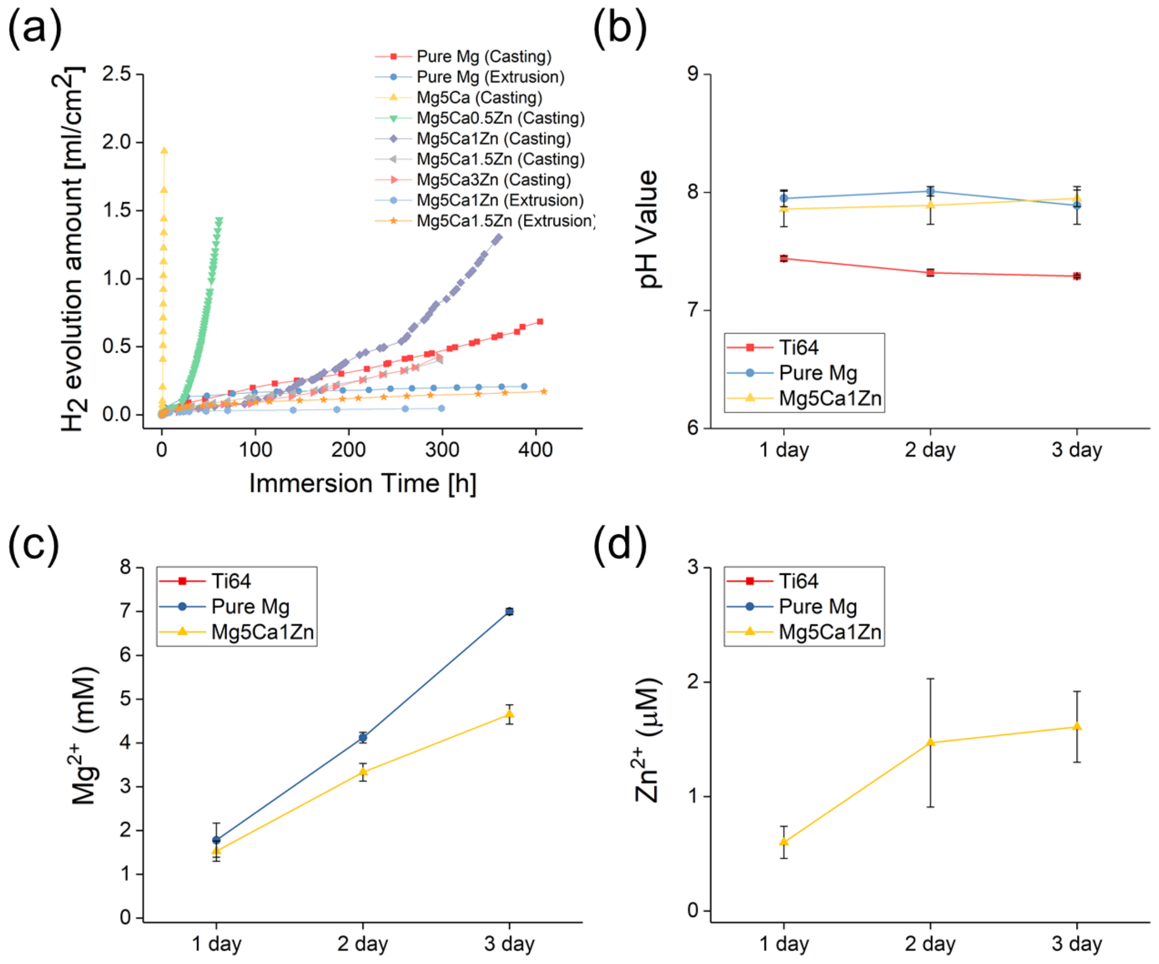

Figure S1. (a) Hydrogen-evolution measurements over time for pure Mg and Mg alloys. (b) Change of pH over time for Titanium alloy, Pure Magnesium and Mg5Ca1Zn. (c) Change of Mg ion concentration over time for Titanium alloy, Pure Magnesium and Mg5Ca1Zn. (d) Change of Zn ion concentration over time for Titanium alloy, Pure Magnesium and Mg5Ca1Zn change.[8, 29]

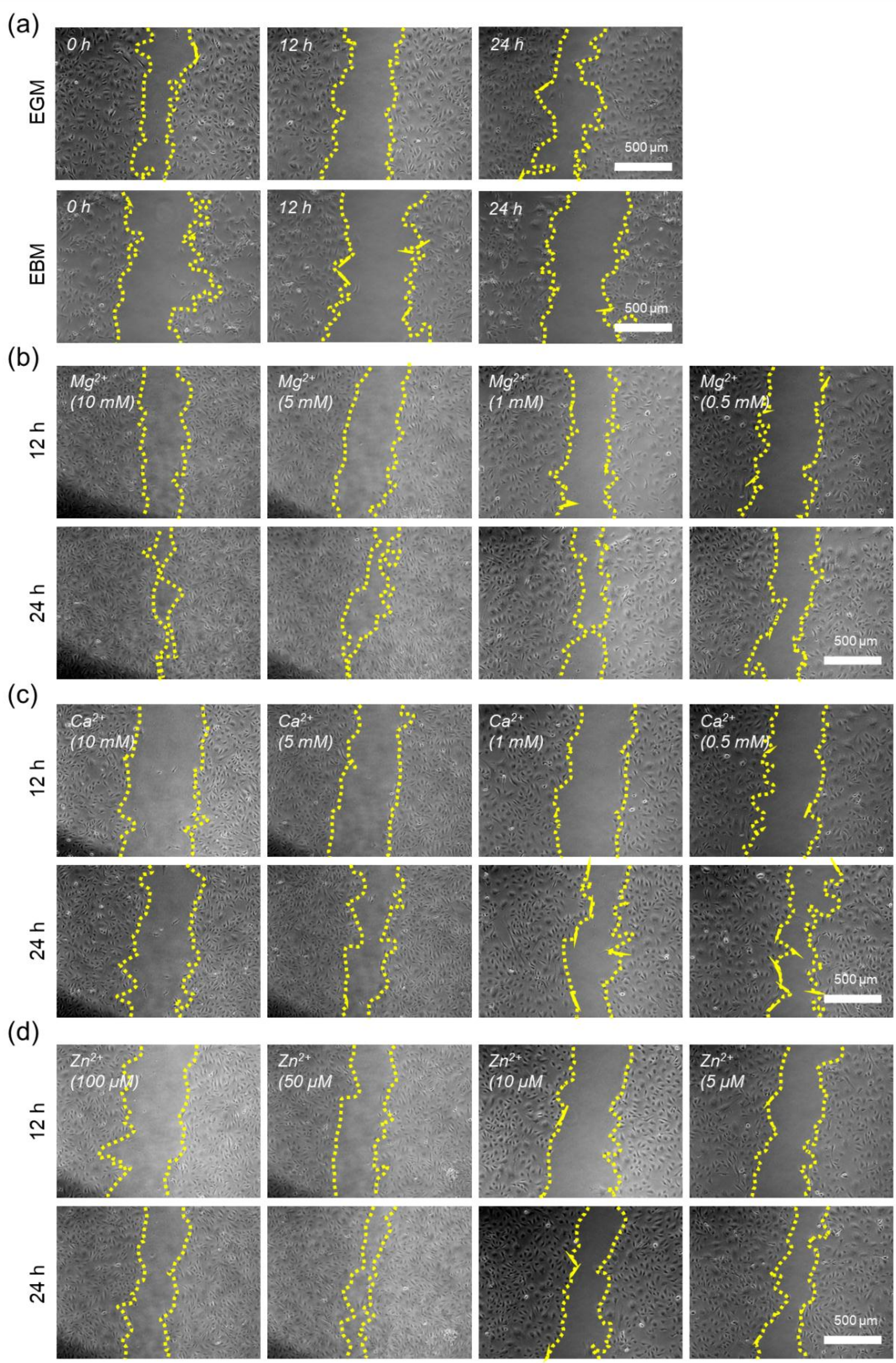

Figure S2. Scratch assay test on HUVEC treated with EGM, EBM, Mg, Ca, Zn ions at 16 h post-injury.

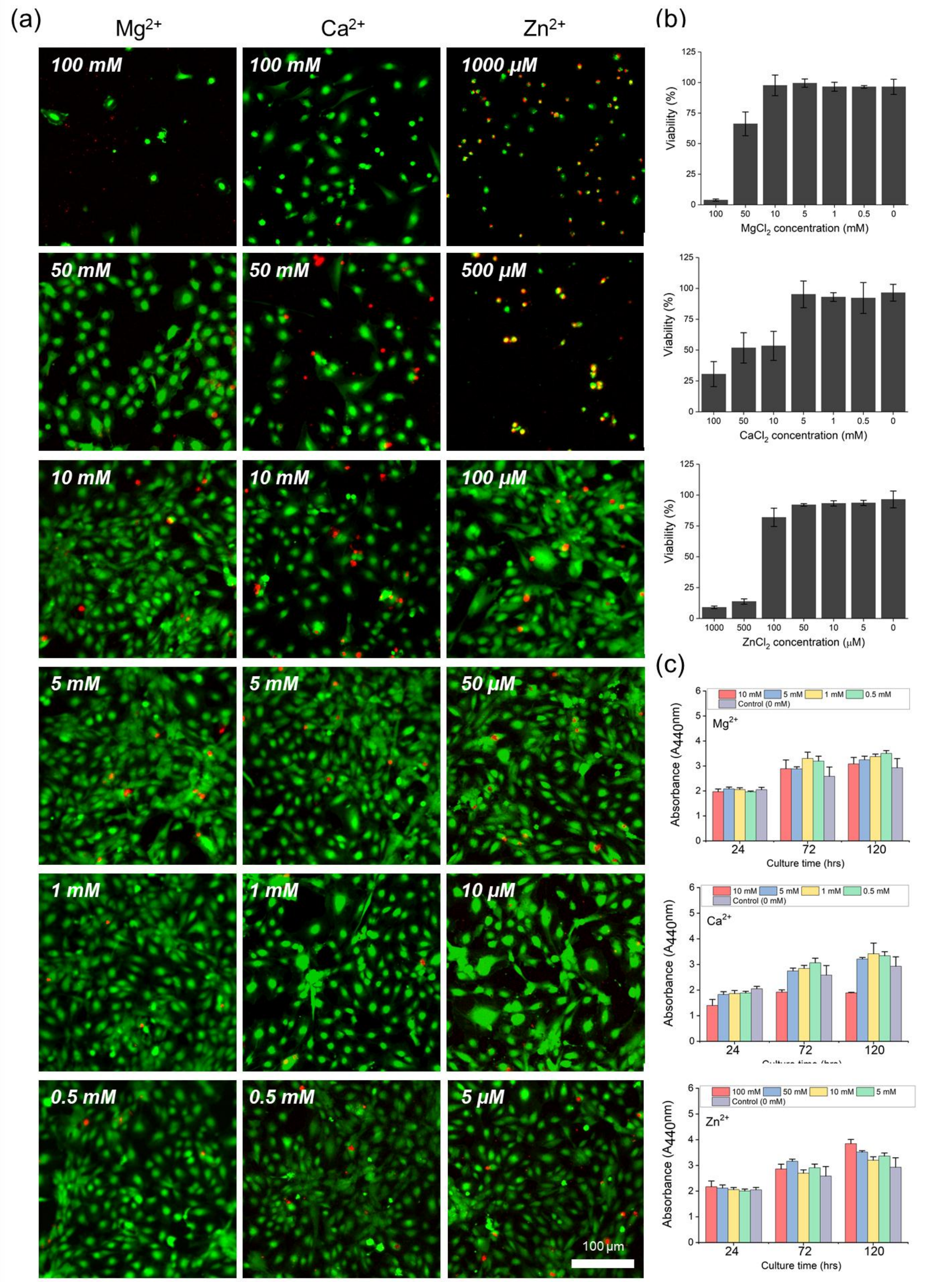

Figure S3. (a) Merged live/dead assay showing cell viability of HUVEC treated with different concentrations of Mg, Ca and Zn ions for 12 h. The image shows live (green) and dead (red) cells simultaneously. (b) Absorbance at 12 h for Mg, Ca and Zn ions. (c) Absorbance throughout culture time for Mg, Ca and Zn ions.

Cell proliferation was evaluated for 120 h using a WST-1 based colorimetric assay (EZ-Cytotox Assay Kit, ITSBIO, Seoul, Korea). As shown in Figure S3c, there is a slight increase in proliferation of HUVEC after 120 h when treated with increasing concentration of Mg ions, but it is not statistically significant. For example, at 120 h, the absorbance values were 3.08 ± 0.25, 3.24 ± 0.14, 3.37 ±0 .10, 3.50 ± 0.11 and 2.92 ± 0.37 for the Mg ion treated at 10, 5, 1, 0.5 mM and control (0 mM), respectively. Cells treated with calcium ions showed decreased proliferation activity with increasing Ca ion concentration. Interestingly, HUVEC treated with increasing concentration of Zn ions showed increasing proliferation activity of HUVEC after 120 h of culture.

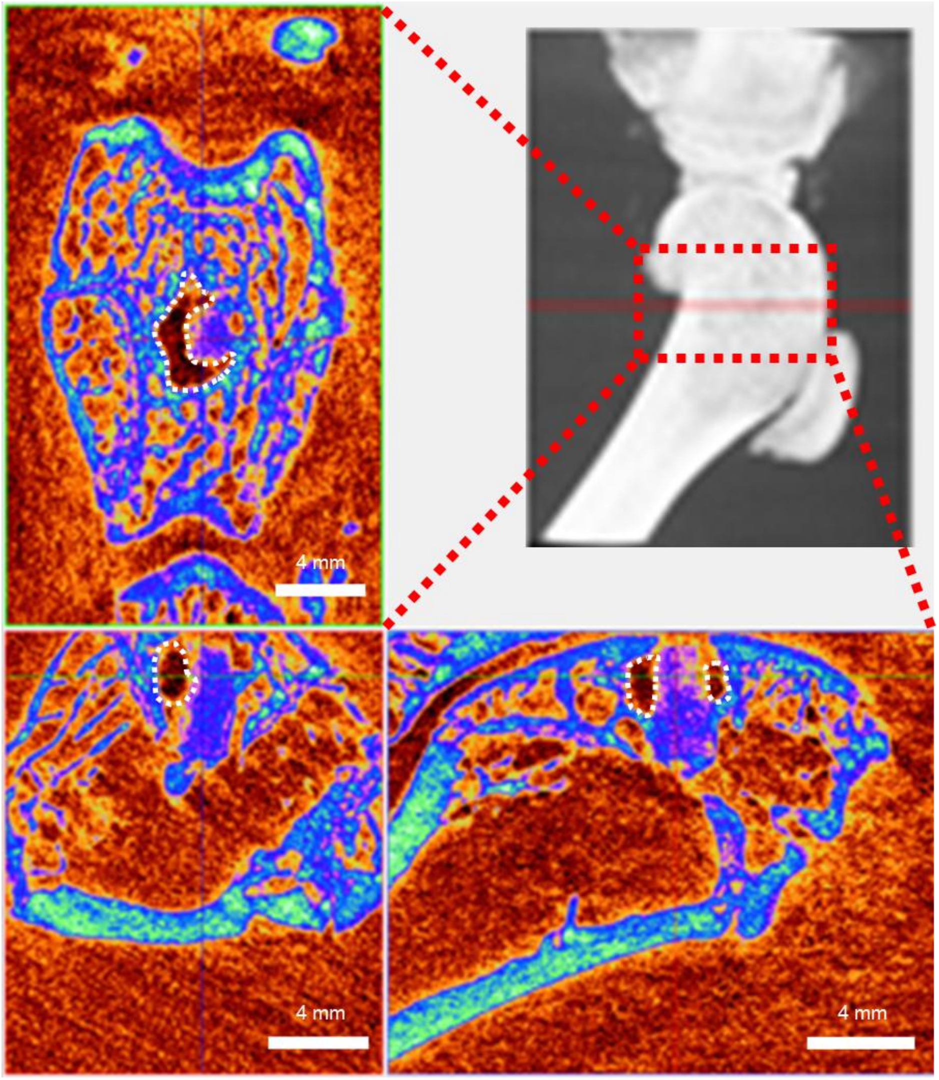

Figure S4. Live µCT image of fast degrading Mg5Ca alloy in SD rats. White dotted area represents low-density tissue next to the implant.

The bone fixation devices must be able to sustain the mechanical loading without causing severe immune response to facilitate the new bone growth at the fracture site. For biodegradable materials, a slow degradation rate is required to allow such integration of the implant to the surrounding tissue. Fast degrading magnesium alloy such as Mg5Ca in the physiological environment, leads to release of hydrogen gas and metallic ions at a rate that is impossible for normal fracture healing to occur. As shown in Figure S4, implantation of fast degrading Mg alloy in the femoral condyle of SD rats resulted in insufficient healing of the bone defect site. It also showed a formation of low-density tissue next to the implant

appearing as an empty space in bone created by the implant. However, it clearly shows darker color when compared to the confirmed empty spaces in the background, suggesting the possibility of diseased fibrotic tissue formation.

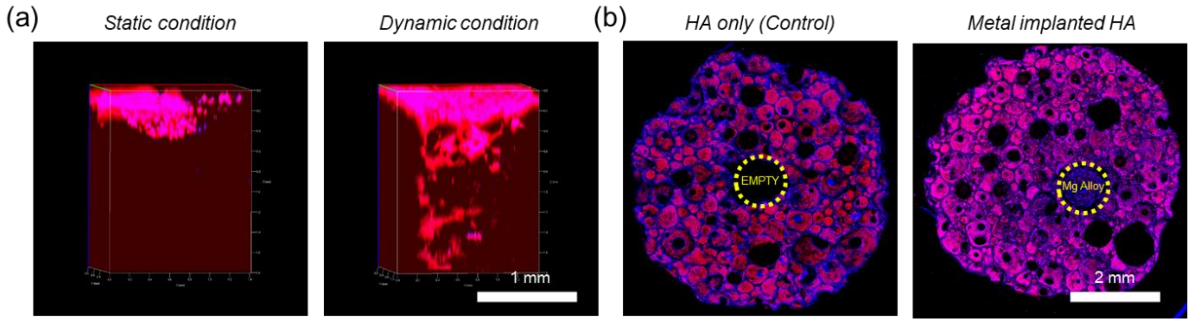

Figure S5. (a) Confocal Z stack image showing human fetal osteoblastic cell proliferation and penetration through 1mm wide and 2 mm deep defect made on HA scaffold under static and perfusion condition after 1 day of culture. (b) Cytoskeletal structures hFOBs after 1 day of culture on the HA control and Metal implanted HA. The images were acquired by fluorescence staining for F-actin (red), and DAPI (nuclei)

The use of a perfusion bioreactor significantly increased the cell penetration through the HA scaffold as shown in Figure S5. The hFOB 1.19 cells did not migrate down to the empty defect site created in HA after 1 day of culture. In contrast, confocal Z stack images showed the migrated hFOB 1.19 cells penetrating the empty defect site of HA in the perfusion conditions after 1 day of culture. Cells showed good attachment to the both HA and Metal implanted HA.

Materials and Methods

Cell culture. An endothelial cell line, Human Umbilical Vein Endothelial Cells (HUVEC), obtained from LONZA group, was grown in Endothelial Cell Growth Medium (EGM) kit in a humidified chamber with 5% CO_2 at 37°C.

Proliferation. Cell proliferation was evaluated for 120 h using a WST-1 based colorimetric assay (EZ-Cytotox Assay Kit, ITSBIO, Seoul, Korea). Briefly, cells were replenished at each assay point (24, 72 and 120 h) with a working solution and incubated for an additional 120 min. The enzymatic activity was then measured at 440 nm using a spectrophotometer. All data were calibrated with an absorbance value calculated using cell-free samples and treated with the same working solution.
